# Social and non-social directional cues differentially orient attention by learned habit

**DOI:** 10.3389/fnhum.2025.1636726

**Published:** 2025-09-09

**Authors:** Claudia Salera, Ala Yankouskaya, Manuel Petrucci, Anna Pecchinenda

**Affiliations:** ^1^SmArt Lab, IRCCS Santa Lucia Foundation, Rome, Italy; ^2^Department of Psychology, Bournemouth University, Poole, United Kingdom; ^3^Department of Human Sciences, Guglielmo Marconi University, Rome, Italy; ^4^Department of Psychology, Sapienza University of Rome, Rome, Italy

**Keywords:** selective attention, spatial orienting, arrow cueing, gaze cueing, learned habit, predictive validity, probability cueing, statistical learning

## Abstract

**Introduction:**

Since an early age, we are implicitly motivated to use the direction of eye gaze of others to learn about the environment, and we orient our attention in space based on this directional signal. Similarly, we orient our attention based on the direction of arrow signs. In both cases, the mechanisms underlying attentional orienting rely on the activity of brain areas involved in endogenous attention; however, orienting by gaze direction also relies on brain areas involved in exogenous attention.

**Research questions:**

To date, it remains unclear whether the acquisition of attentional habit, which can also guide attention in ways that are not purely endogenous or exogenous, is similar for gaze and arrow or rather differs in some important way. We aimed to assess whether learning implicit regularities implemented with exogenous, arrow, and gaze stimuli guides attention in space.

**Methods:**

Using the Posner paradigm, we conducted a series of behavioral experiments with exogenous, arrow, and gaze cues. Unbeknownst to participants, specific regularities, namely cue predictive validity and probability cueing, were implemented through blocks (baseline, learning, testing).

**Results and discussion:**

The findings showed that predictive validity alone is not sufficient to engender habitual attention for all types of cues. However, it becomes effective when combined with probability cueing. Importantly, a learned habit with gaze cues engenders unique effects on attention compared to other cues.

**Conclusion:**

Socially relevant directional signals, such as gaze, can bias spatial attention more effectively than perceptual or non-social directional stimuli.

## 1 Introduction

We live in a rich environment, and we cannot pay attention to all the information; therefore, selection is necessary. Traditional theoretical and neuroscientific perspectives propose that selective attention works through endogenous (goal-directed) or exogenous (stimulus-driven) mechanisms (Corbetta and Shulman, 2002). This view has been extended (Pourtois et al., 2013) and challenged by the observation that our cognitive system can detect environmental regularities and can learn to use them for attentional deployment (Ferrante et al., 2018). That is, attention can also be guided by experience, which can induce habit formation and an attentional bias. Interestingly, attention can be guided by a learned habit that challenges the traditional dichotomy between exogenous and endogenous mechanisms. Indeed, Awh et al. (2012) conceptualized attention as resulting from priority maps, where different mechanisms all contribute to affect attention. Therefore, learning implicit environmental regularities can modulate attention in a way that shares characteristics of both exogenous and endogenous attention.

The effect of habit formation on attention has been shown with the visual search task. If relevant events (i.e., targets) are more frequent at one spatial location (i.e., the rich location) than at others (i.e., scarce locations), an attentional bias for the rich location occurs. Thus, participants direct their attention more rapidly to the rich location (Addleman and Lee, 2022), and they respond more accurately and rapidly to targets that appear at the rich location compared to targets that appear at the scarce locations. This effect is known as location probability learning (also referred to as probability cueing; Geng and Behrmann, 2002; Jiang et al., 2013; Wang and Theeuwes, 2018). In addition, this attentional preference for the rich location persists also when the regularity is removed, and targets occur with the same frequency at all spatial locations (Jiang, 2018).

In the Posner cueing paradigm (Posner, 1980), regularities can be induced by increasing cue predictive validity (i.e., the ability of the cue to predict where the target appears) and by probability cueing (the frequency at which targets appear at one location). More specifically, in a variant of this paradigm, a peripheral cue (e.g., a change in luminance) is presented left or right of a central fixation (e.g., a plus sign), and the to-be-detected target can appear either at the location validly cued or at the location invalidly cued. When peripheral cues are not predictive (i.e., 50% cue predictive validity), the typical finding is that responses are faster and more accurate to valid trials compared to invalid trials. The difference in response time between the two conditions (i.e., valid and invalid) is known as the “cueing effect.” The “advantage” produced by valid cues is due to attention being already at the location indicated by the cue when the target appears. In contrast, the “cost” produced by invalid cues is due to having to disengage attention from the invalidly cued location and reorient it to the target location. As cueing effects with peripheral cues occur rapidly, at short (e.g., 100–250 ms) Stimulus Onset Asynchronies (SOAs) and with cues that are not predictive of target location, orienting of attention is considered involuntary and exogenous. Importantly, when exogenous cues have predictive validity, the cueing effect is larger (Risko and Stolz, 2010; López-Ramón et al., 2011; Gough et al., 2014; Lanthier et al., 2015).

Similar effects are also observed in another variant of the Posner paradigm, where a directional cue (i.e., arrow-cue or gaze-cue) is presented in the center of the screen, indicating left or right, and the target to be detected appears either at the validly cued or invalidly cued location. Although orienting attention with central, symbolic cues is typically described as a voluntary mechanism because it takes time, it occurs only at longer (>300 ms) SOAs (Müller and Rabbitt, 1989), and it requires the cues to predict the target. Evidence shows that arrows (Eimer, 1997; Tipples, 2002) and gaze (Friesen and Kingstone, 1998) cues orient attention in a way that shares some characteristics of exogenous orienting. Namely, orienting occurs with short SOAs, with cues that are non-predictive of target location, and with participants being aware that using the cue direction to orient their attention has no advantages (Chica et al., 2014). Importantly, it is still debated whether orienting by these two types of cues relies on similar mechanisms (Friesen et al., 2004) and neural substrates (Chacón-Candia et al., 2023; Salera et al., 2024). A common assumption is that when arrows and gaze reliably predict where the target appears, voluntary mechanisms of orienting spatial attention are recruited (Carrasco, 2011). However, arrow and gaze have also been found to produce greater cueing effects than reflexive and volitional orienting (Ristic and Kingstone, 2006). This evidence has been attributed to the intrinsic perceptual asymmetry of these stimuli and to their overlearned directional value due to environmental and social exposure (Ristic and Kingstone, 2012).

In the Posner paradigm, larger cueing effects are observed when cues have predictive validity, suggesting that we can detect and learn from regularities present in the environment and use them to improve our performance. However, it is unclear whether this learning results in habit formation in similar ways for exogenous, arrow, and gaze cues and whether habitual attention persists once the regularities are no longer in place. This research question arises from evidence suggesting that learning regularities with social cues may be different from learning regularities with non-social cues. In fact, we detect regularities in the gaze direction of others and we acquire preferences based on these regularities (Bayliss et al., 2006; Tipples and Pecchinenda, 2019). In addition, gaze direction is a relevant source of information since infancy, as it facilitates object processing (Michel and Thiele, 2025) and earlier attentional orienting compared to arrow cues (Jakobsen et al., 2013; Ishikawa and Yoshioka, 2025). Neuroimaging data also highlight the differences between orienting attention by social and non-social cues, as processing gaze direction relies on brain areas involved in mental state attribution, such as the superior temporal sulcus and temporoparietal junction (Salera et al., 2024). Since we recognize that people focus on what matters most to them, our intrinsic motivation to learn from this directional signal may be stronger than our motivation to learn from non-social directional signals. However, whereas brain regions involved in endogenous attention underlie orienting attention by social and non-social cues, the medial frontal gyrus involved in exogenous attention plays a role only in orienting attention by gaze direction (Salera et al., 2024). What remains unclear is whether the detection of regularities with gaze and arrow cues leads to the kind of habitual attentional biases observed with non-symbolic exogenous cues, raising the question of whether similar mechanisms underlie learning across social and non-social signals. Therefore, in a series of seven experiments using exogenous, arrow, and gaze cues, we assessed whether implicit regularities implemented by varying cue predictive validity alone or in combination with probability cueing are learned and induce habit formation that can guide attention. If gaze direction has special relevance for us compared to non-social directional stimuli or compared to exogenous stimuli, then learning regularities with gaze cues may induce an attentional habit in ways that differ from those resulting from learning regularities with non-social cues. In a previous study using the gaze cueing task, we found that older adults who typically show reduced attentional orienting with non-predictive gaze cues can detect and learn from gaze regularities, which enhanced the gaze cueing effects (Salera et al., 2024). These findings suggest that combining social cues with relevant environmental events may be crucial for acquiring a habit that guides attention; however, they cannot speak to whether such habit formation is similar for non-social and for exogenous cues. In addition, Salera et al. (2024) demonstrated that cue predictive validity varied in conjunction with probability cueing, and it remains unclear whether learning involves the ability of the cue to predict the target and the probability of the target’s appearance at a particular location. This ambiguity leaves open the question of whether predictive learning with different cue types (social, symbolic, or exogenous) relies on shared or distinct mechanisms, and whether such learning supports the formation of enduring attentional habits. In seven experiments, we investigated whether regularities with exogenous, arrow, and gaze cues are learned, resulting in a habit that guides attention. We varied cue predictive validity alone (Experiments 1, 3, and 5) or in combination with target location probability (Experiments 2, 4, and 6). We anticipated that if regularities are learned, they should guide attention even after they are no longer in place (i.e., in the testing phase). We implemented the same regularities in all cueing experiments, and we predicted that, considering that we learn from gaze direction since infancy, learning regularities with social and non-social cues may result in different patterns of habitual attention. Thus, considering the extensive experience in learning social cues, habitual attention with gaze cues may resemble the pattern of habitual attention with exogenous cues.

## 2 Methods

We report how we determined our sample size, all data exclusions (if any), all manipulations, and all measures in the studies. Data is available at the Open Science Framework (OSF) at the following link: https://osf.io/w7ynj/?view_only=5c7be7a84088400480268802d5f5330e.

### 2.1 Participants

A total of 322 undergraduate students volunteered to take part in one of the seven experiments in exchange for course credit (267F, mean age in years: 22.04; SD: 2.88). The data of four participants in Experiment 1 (exogenous cues, no bias) were excluded from the analyses due to technical issues. The resulting sample consisted of *N* = 318 (263F, mean age in years: 22.05; SD: 2.89). Table 1 shows the sample characteristics for each experiment.

**Table 1:** Characteristics of the sample for each experiment.

Experiment	Sample N	Gender	Age in years M (SD)
1: exogenous cues no bias	41	36F	21.34 (1.69)
2: exogenous cues with bias	45	38F	21.13 (1.83)
3: arrow cues no bias	51	45F	22.92 (4.84)
4: arrow cues with bias	45	37F	24.33 (2.68)
5: gaze cues no bias	45	39F	21.29 (1.61)
6: gaze cues with bias	45	37F	21.36 (1.76)
7: targets with bias	46	30F	21.83 (2.57)

We calculated the required sample size using effect sizes from previous research with a similar experimental design, applying G*Power (Faul et al., 2007). To achieve 95% power with an alpha level of <0.05 and an effect size of *f* = 0.25 in a repeated-measure, within-factors ANOVA, each experiment requires a minimum of 28 participants. All participants provided informed consent, which was obtained in accordance with the Declaration of Helsinki (World Medical Association, 2013). The study received approval from the institutional review board (approval number 0000867, dated April 28, 2021). Participants had normal or corrected-to-normal vision and were unaware of the purpose of the study.

### 2.2 Stimuli and apparatus

For all tasks, two black rectangles (the lines were 2 pixels in width) measuring 2.6 cm in height and 2 cm in width served as placeholders. They were placed 53 mm to the left and right of the central fixation, which was a cross measuring 0.3 by 0.3 cm. The targets were an “L” and a “T” (0.9 cm vertically and 0.6 cm horizontally). For Experiments 1 and 2, the (exogenous) cue was a change in luminance (from black to white) of one placeholder. For Experiments 3 and 4, the cue was a central arrow pointing either left or right. For Experiments 5 and 6, the cue was a schematic face with eyes looking either to the left or right. The arrow was created by combining a straight, horizontal line (3 cm, 0.3 in thickness) with an arrow tail and an arrowhead (1.0 cm × 1.0 cm). The schematic face consisted of a round black line of 7 cm in diameter and 0.3 cm in thickness. The eyes were two black line circles measuring 1.5 cm in diameter and 0.3 cm line thickness. Pupils consisted of two black-filled circles, 0.7 cm in diameter, positioned on the left or right side of the eyes, as described by Hietanen et al. (2006).

The task was presented using E-Prime Version 3.0 software (Psychology Software Tools, Pittsburgh, PA, 2012). All stimuli were presented on a gray background against a 19-inch LCD monitor (resolution 1920 × 1,080, refresh rate 60 Hz). Responses were collected using a standard USB keyboard.

### 2.3 Procedure

Upon arrival at the laboratory, participants provided their written informed consent, following which they were invited to a dimly lit room where they sat comfortably in front of a computer screen, at a viewing distance of approximately 60 cm (ensured using a chinrest). Participants were instructed to maintain fixation and respond as fast and as accurately as possible to the target letter L or T based on its identity. To avoid left–right response mapping, they responded by pressing one of two adjacent keys, chosen to be perpendicular to the left and right target positions (“1” and “5” keys), accordingly labeled. The key assignment to targets was counterbalanced between participants.

**Figure 1:**
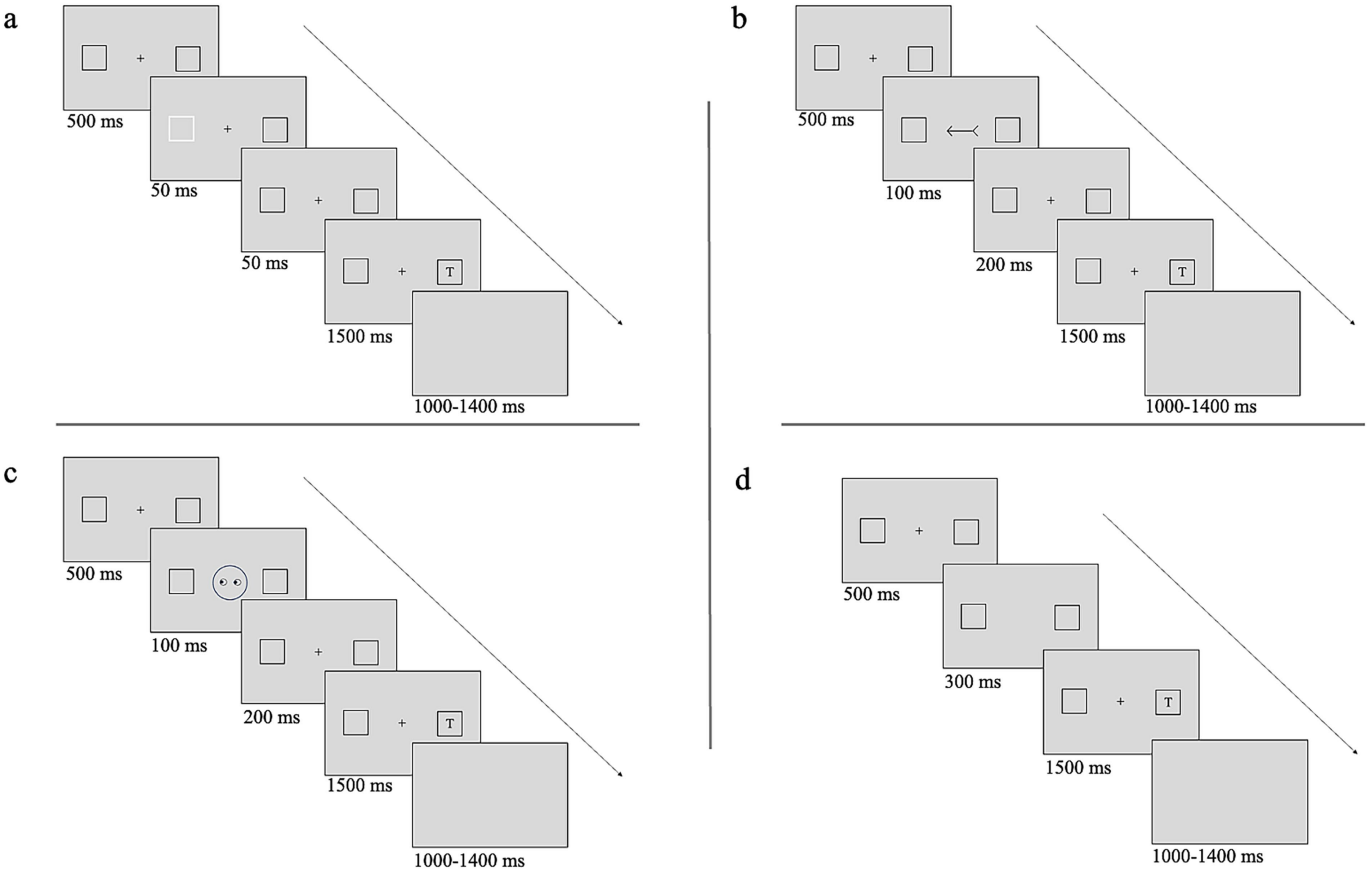
Sequence of events in a typical trial. The example shows a trial with **(a)** the target following an invalid exogenous cue, **(b)** the target following an invalid arrow-cue, **(c)** the target following an invalid gaze-cue, or **(d)** the target alone.

For Experiments 1 and 2 with exogenous cues, each trial began with the display of two peripheral placeholders, located to the left and right of the central fixation, for 500 ms, followed by the cue for 50 ms (i.e., one placeholder brightened). After a SOA of 100 ms, chosen to enhance rapid exogenous cueing effects (Risko and Stolz, 2010; Gough et al., 2014; Chica et al., 2014; Meijs et al., 2018), the target (L or T) appeared in the center of one of the placeholders and remained on screen until response or 1500 ms had elapsed (see Figure 1a). For Experiments 3, 4, 5, and 6 with central cues (arrow and gaze), each trial started with the display of two peripheral placeholders left and right relative to the central fixation for 500 ms, followed by the cue consisting of an arrow pointing left or right (see Figure 1b) or a schematic face looking left or right (Figure 1c) for 100 ms. After a SOA of 300 ms, chosen to enhance cueing effects (McKay et al., 2021; Langdon and Smith, 2005), the target (L or T) appeared in the center of one of the placeholders. The target remained on screen until a response or 1500 ms had elapsed. Finally, for Experiment 7 with target only, each trial began with the display of two peripheral placeholders, left and right, relative to the central fixation (500 ms). After 300 ms, the target (L or T) appeared in the center of one of the placeholders (see Figure 1d). The target remained on screen until a response or 1500 ms had elapsed. To prevent strategies based on temporal expectancies (Meijs et al., 2018), the Inter-Trial Interval (ITI) varied randomly between 1000 and 1400 ms in steps of 100 ms.

In each cueing experiment, after 32 practice trials with cue predictive validity at 50% (i.e., non-predictive cues), there were 560 trials divided into five blocks of 112 trials each. Between blocks, participants had the opportunity to take a short break. Unbeknownst to participants, cue predictive validity started at 50% in Block 1 (Baseline), increased to 75% (i.e., predictive cues) in Blocks 2 to 4 (Learning), and returned to 50% in Block 5 (Testing). Table 2 depicts the number of trials with valid and invalid cues in the three phases. The allocation of trials with valid and invalid cues at the two spatial locations in the experiments with biased location was derived from the 84 trials with valid cues and the 28 trials with invalid cues used in the experiments with unbiased location. To implement the location bias, the 84 trials with valid cues were asymmetrically distributed between the rich and scarce location as follows: (a) 75% of the 84 trials with valid cues (i.e., 63 trials, rounded up to 64 as odd numbers dop not allow to have an equal number of the two target-letters) were assigned to the rich location; (b) the remaining 25% of 84 trials with valid cues (i.e., 21 trials, rounded down to 20) were assigned to the scarce location. Similarly for the 28 trials with invalid cues of the unbiased condition: (a) 75% of the 28 trials with invalid cues (i.e., 21 trials, rounded up to 22) were allocated to the rich location; (b) the remaining 25% of 28 trials with invalid cues (i.e., 7 trials, rounded down to 6) were allocated to the scarce location. Assignment of left or right to the rich location was counterbalanced across participants.

**Table 2:** Distribution of trials with valid and invalid cues at the two spatial locations in experiments 1, 3, and 5 with no (location) bias and in Experiments 2, 4, and 6 with (location) bias.

Phases	Baseline (1 block × 112 trials) 50% cue predictive validity		Learning (3 blocks × 112 trials) 75% cue predictive validity		Testing (1 block × 112 trials) 50% cue predictive validity	
Proportion validity	½ valid	½ invalid	¾ valid	¼ invalid	½ valid	½ invalid
Number of trials	56 trials	56 trials	84 trials	28 trials	56 trials	56 trials
No bias	28 left 28 right	28 left 28 right	42 left 42 right	14 left 14 right	28 left 28 right	28 left 28 right
With bias	28 rich 28 scarce	28 rich 28 scarce	64 rich 20 scarce	6 rich 22 scarce	28 rich 28 scarce	28 rich 28 scarce

Assignment of the “Rich” and “Scarce” condition to the left or right location was counterbalanced across participants.

### 2.4 Experimental design and data analyses

For the cueing experiments, a 3 (phase: baseline, learning, testing) by 2 (Cue: valid, invalid) by 2 (Location: left, right in experiments without location bias; rich, scarce in experiments with location bias) within-subject design was used. For the target-only experiment, a 3 (phase: baseline, learning, testing) by 2 (Location: rich, scarce) within-subject design was used.

For each experiment, reaction times (RT) from trials with errors, RTs below 120 ms, and outliers (RTs above 2.5 SD of the overall mean) were excluded from the analyses. Experiment 1: trials with errors = 1.86%, <120 ms = 0.13; outliers = 4.3%; Experiment 2: trials with errors = 2.04%, <120 ms = 0.13%; outliers = 2.04%; Experiment 3: trials with errors = 4.97%, <120 ms = 0.67%; outliers = 7.83%; Experiment 4: trials with errors = 3.23%, <120 ms = 0.3%; outliers = 6.07%; Experiment 5: trials with errors = 5.07%, <120 ms = 0.23%; outliers = 7.67%; Experiment 6: trials with errors = 2.98%, <120 ms = 0.27%; outliers = 5.76%; Experiment 7: trials with errors = 4.31%, <120 ms = 0.14%; outliers = 6.84%. Next, for RTs and response accuracy, the means for each condition were computed. A cueing index was computed for each phase based on the relative change in overall response speed according to the following formula:


[(RT_Invalid–RT_Valid)/(RT_Invalid+RT_Valid)/2]∗100.

This process of calculating relative difference scores is important when making quantitative comparisons between groups and/or across blocks (e.g., Ramon et al., 2010; Salera et al., 2024).

The cueing index was analyzed using a repeated-measures ANOVA with phase (3: baseline, learning, testing) by Location (2: left vs. right or rich vs. scarce, depending on the experiment). Data (RTs and accuracy) for the target-only experiment were analyzed using a 3 × 2 repeated measures ANOVA with phase and location as factors. When the interaction was statistically significant, comparisons examined changes across the 3 phases (baseline, learning, testing) for each location to determine whether learning had occurred and persisted once the contingencies were removed. All pairwise comparisons were corrected for multiple comparisons using the Bonferroni method (complete ANOVAs for RTs and accuracy are reported in Supplementary material). In addition to traditional frequentist statistics (e.g., *p*-values), Bayes Factors (BF10) were calculated to complement the interpretation of relevant effects. Bayes Factors quantify the strength of evidence for the alternative hypothesis (BF_10_ > 1). A Bayes Factor above 3 is considered moderate evidence, and above 10 is considered strong evidence in favor of the alternative hypothesis.

## 3 Results

### 3.1 Experiments 1 and 2 with exogenous cues

**Figure 2:**
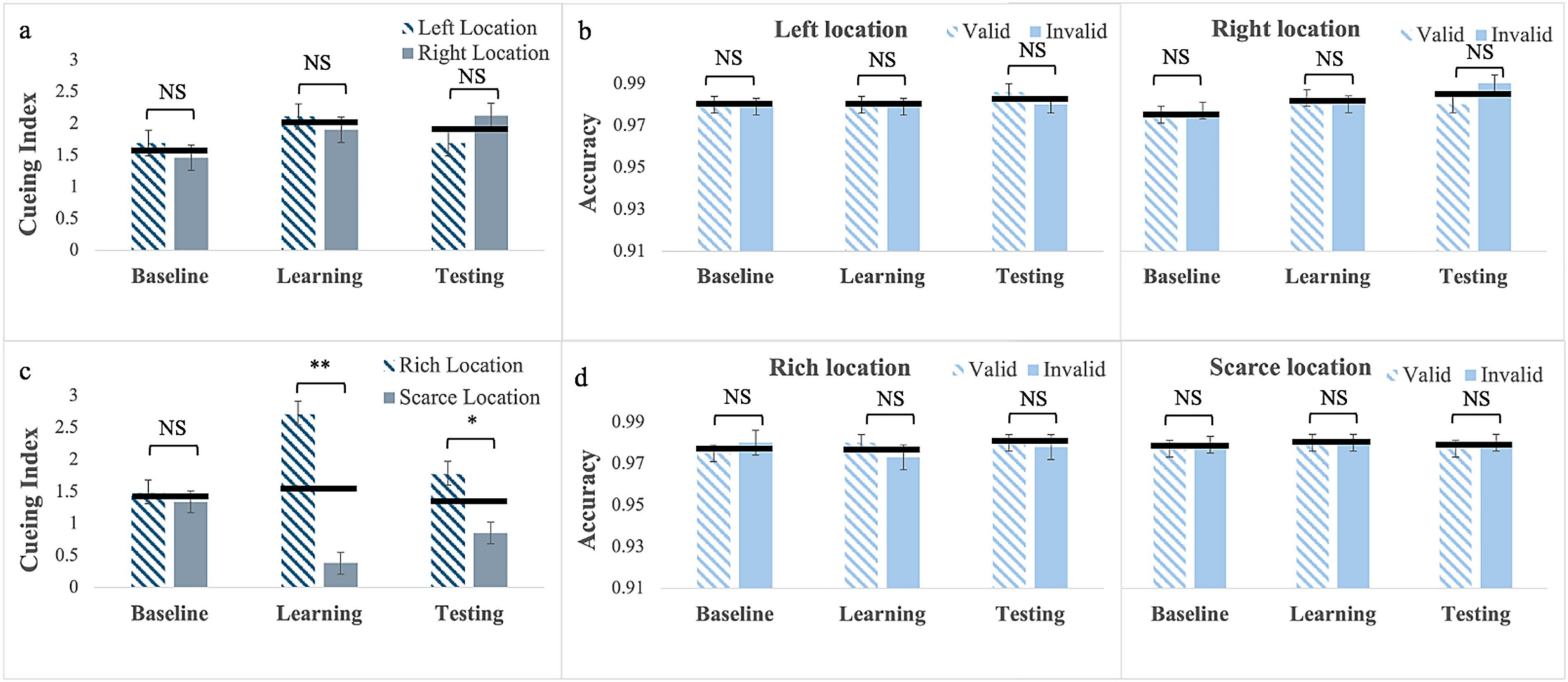
Cueing index **(a,c)** and response accuracy **(b,d)** across the three experimental phases (Baseline, Learning, Testing) for Experiment 1 with exogenous cues with no location bias (**a,b**, top row) and for Experiment 2 with exogenous cues with location bias (c and d, bottom row). Error bars are standard errors of the mean. Asterisks denote significant differences: one asterisk (*) for *p* < 0.05; two asterisks (**) for *p* < 0.001. Black horizontal bars denote average values for each pair.

*Cueing Index*: For exogenous cues without location bias (exp. 1), Mauchly’s Test of sphericity was significant for phase, W = 0.76; χ^2^(2) = 10.56, *p* = 0.005, and for phase by location, W = 0.72; χ^2^(2) = 12.61, *p* = 0.002; therefore, we report Greenhouse–Geisser corrected values. There was a significant main effect of phase, *F*_(1.62, 64.66)_ = 4.21, *p* = 0.026, partial η^2^ = 0.095. Pairwise comparisons showed that the cueing index in the learning (M = 2.01; SE = 0.14) was larger than in the baseline (M = 1.58; SE = 0.15), *p* = 0.01 but it did not differ from testing (M = 1.90; SE = 0.16), *p* = 0.28. The main effect of Location, *F*_(1, 40)_ < 0.005, *p* = 0.99 and the phase by location interaction, *F*_(1.57, 62.68)_ = 2.32, *p* = 0.118 were not statistically significant (see Figure 2a).

*Response Accuracy*: the Mauchly’s Test of sphericity was significant for phase by cue, W = 0.65; χ^2^(2) = 16.56, *p* < 0.001, for phase by location, W = 0.85; χ^2^(2) = 6.49, *p* = 0.039, and for the 3-way interaction, W = 0.62; χ^2^(2) = 18.44, *p* < 0.001. Therefore, we report Greenhouse–Geisser corrected values. There was a significant main effect of phase *F*_(2, 80)_ = 3.61, *p* = 0.032, partial η^2^ = 0.083, but pairwise comparisons failed to show any significant difference (see Figure 2b). No other main effect or interactions reached statistical significance (see Supplementary material).

*Cueing Index*: For exogenous cues with location bias (Experiment 2), the results showed that the main effect of phase was not statistically significant, *F*_(2, 88)_ = 1.20, *p* = 0.307. The main effect of Location was significant, *F*_(1, 44)_ = 28.80, *p* < 0.001, partial η^2^ = 0.40 with a larger cueing effect at the rich (M = 1.99; SE = 0.13) than at the scarce location (M = 0.86; SE = 0.13). This was qualified by a significant phase by location interaction, *F*_(2, 88)_ = 18.56, *p* < 0.001, partial η^2^ = 0.30. Pairwise comparisons showed that for the rich location cueing was greater in the learning (M = 2.71; SE = 0.18) than in the baseline, (M = 1.48; SE = 0.21) *p* < 0.001, cueing was still numerically greater in the testing phase (M = 1.77; SE = 0.22) than in the baseline, but the difference was not statistically significant, *p* = 0.97. For the scarce location, the reverse pattern was present with a smaller cueing in the learning (M = 0.38; SE = 0.17) than in the baseline (M = 1.34; SE = 0.17), *p* < 0.001. Again, although cueing was still numerically smaller in testing (M = 0.85; SE = 0.18) than in the baseline, the difference was not statistically significant, *p* = 0.11. Finally, Bayesian analyses showed moderate evidence (BF_10_ = 3.60) that cueing for the scarce location was greater in the testing than in the learning phase, *p* = 0.03 (see Figure 2c). This pattern resulted in no cueing differences in the baselines, *p* = 0.60, very strong evidence (BF_10_ > 100) of larger cueing for the rich location in the learning, *p* < 0.001, and moderate evidence (BF_10_ = 4.55) of larger cueing for the rich location for testing, *p* = 0.008.

*Response Accuracy*: Results showed no significant main effects or interactions (see Figure 2d; Supplementary material).

To sum up, for exogenous cues, increasing predictive validity alone did not induce an attentional habit that persisted to the testing phase (Experiment) In contrast, when paired with probability cueing (Experiment 2), learning induced habitual attention, which consisted of an advantage for the rich location (i.e., larger cueing effects) and a cost (smaller cueing effects) for the scarce location. This pattern was evident during the learning phase and continued into the testing phase.

### 3.2 Experiments 3 and 4 with arrow cues

**Figure 3:**
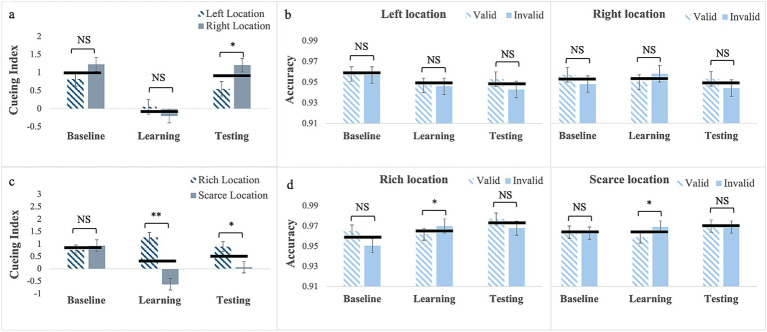
Cueing index **(a,c)** and response accuracy **(b,d)** across the three experimental phases (Baseline, Learning, Testing) for Experiment 1 with arrow cues with no location bias (**a,b**, top row) and for Experiment 2 with arrow cues with location bias (**c,d**, bottom row). Error bars are standard errors of the mean. Asterisks denote significant differences: one asterisk (*) for *p* < 0.05; two asterisks (**) for *p* < 0.001. Black horizontal bars denote average values for each pair.

*Cueing Index*: For arrow cues without location bias (Experiment 3), results showed a significant main effect of phase, *F*_(2, 100)_ = 18.82, *p* < 0.001, partial η^2^ = 0.27. Pairwise comparisons showed that cueing was smaller in the learning phase (M = −0.07; SE = 0.12) than in the baseline (M = 1.02; SE = 0.13) *p* < 0.001 and testing (M = 0.88; SE = 0.18), *p* < 0.001 phases. Cueing in the baseline and testing did not differ, *p* = 0.99. The main effect of Location, *F*_(1, 50)_ = 3.03, *p* = 0.09, and the 2-way interaction *F*_(2, 100)_ = 3.05, *p* = 0.052, were not statistically significant (see Figure 3a).

*Response Accuracy*: There were no statistically significant main effects or interactions (see Figure 3b; Supplementary material).

*Cueing Index*: For arrow cues with location bias (exp.4), Mauchly’s Test of sphericity was significant for phase, W = 0.86; χ^2^(2) = 6.33, *p* = 0.04, and for phase by location, W = 0.78; χ^2^(2) = 10.45, *p* = 0.005. Therefore, we report Greenhouse–Geisser corrected values. The main effect of phase was significant, *F*_(1.76, 77.41)_ = 4.07, *p* = 0.025, partial η^2^ = 0.085; however, after correcting for multiple comparisons, pairwise comparisons failed to show any significant differences. The main effect of Location was significant, *F*_(1, 44)_ = 15.03, *p* < 0.001, partial η^2^ = 0.255, with larger cueing at the rich (M = 0.98; SE = 0.13) than at the scarce location (M = 0.13; SE = 0.14). This effect was further qualified by a significant 2-way interaction, *F*_(1.65, 72.38)_ = 10.69, *p* < 0.001, partial η^2^ = 0.20. Differently from what observed with exogenous cues (Experiment 2), for the rich location, pairwise comparisons showed no differences in cueing across the three phases (baseline: M = 0.76; SE = 0.18; learning: M = 1.23; SE = 0.19, testing: M = 0.90; SE = 0.23). In contrast, for the scarce location, cueing was smaller in the learning phase (M = −0.62; SE = 0.25) than in the baseline phase (M = 0.94; SE = 0.24), *p* < 0.001 and it was greater in the testing phase (M = 0.07; SE = 0.21) than in the learning phase, *p* = 0.02) in this case the BF_10_ = 4.908 indicates moderate evidence. Cueing in the testing phase was still smaller than in the baseline, *p* = 0.04 (see Figure 3c), and the BF_10_ = 3.307 indicates moderate evidence. This pattern resulted in no differences in the baseline, *p* = 0.58, and in significant differences in cueing between the two locations in the learning, *p* < 0.001 (with a BF_10_ > 100, indicating strong evidence) as well as in the testing phase, *p* = 0.03 (with a BF_10_ = 1.57, indicating weak evidence).

*Response Accuracy*: Mauchly’s test of Sphericity was significant for phase, W = 0.78; χ^2^(2) = 10.56, *p* = 0.005, and for phase by cue, W = 0.74; χ^2^(2) = 12.72, *p* = 0.002; therefore, we report Greenhouse–Geisser corrected values. The phase by cue interaction was statistically significant, *F*_(1.59, 68.18)_ = 3.54, *p* = 0.05, partial η^2^ = 0.08. Pairwise comparisons showed no differences in response accuracy between trials with valid (M = 0.97; SE = 0.01) and invalid (M = 0.96; SE = 0.01) cues for the baseline, *p* = 0.22 and between trials with valid (M = 0.97, SE = 0.01) and invalid (M = 0.97, SE = 0.01) cues in the testing, *p* = 0.23. In contrast, in the learning phase, response accuracy was lower on trials with valid (M = 0.96, SE = 0.01) than invalid (M = 0.97, SE = 0.01) cues, *p* = 0.002 (see Figure 3d). That regularities had a negative impact on response accuracy suggests a temporary monitoring adjustment necessary to optimize performance following changes (from the baseline) in predictive validity and target probability appearance at a certain location (Beesley et al., 2015; Walker et al., 2019). In fact, in the testing phase, response accuracy exhibits a typical pattern with greater accuracy on trials with valid cues than on trials with invalid cues, especially at the rich location.

To sum up, similarly to what was observed with exogenous cues in Experiment 1, learning based on predictive validity alone did not yield habitual attention for arrow cues. In fact, cueing effects were smaller in the learning phase, but this effect did not persist to the testing phase. When combined with probability cueing, learning the regularities with arrow cues yields habitual attention. However, in contrast to what was observed with exogenous cues in Experiment 2, this consisted only of a cost (i.e., smaller cueing) for the scarce location.

### 3.3 Experiments 5 and 6 with gaze cues

**Figure 4:**
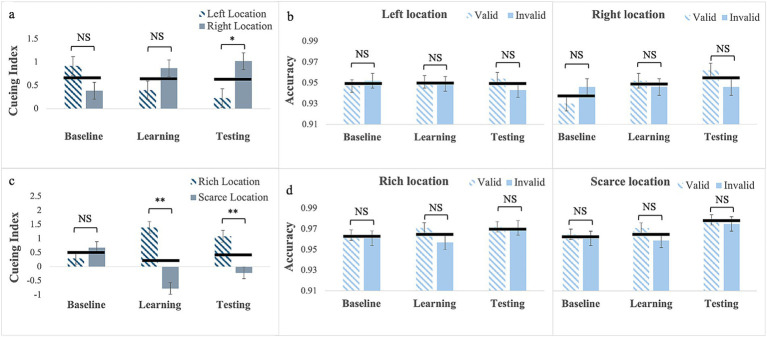
Cueing index **(a,c)** and response accuracy **(b,d)** across the three experimental phases (Baseline, Learning, Testing) for Experiment 1 with gaze cues with no location bias (**a,b**, top row) and for Experiment 2 with gaze cues with location bias (c and d, bottom row). Error bars are standard errors of the mean. Asterisks denote significant differences: one asterisk (*) for *p* < 0.05; two asterisks (**) for *p* < 0.001. Black horizontal bars denote average values for each pair.

*Cueing Index*: For gaze cues without location bias (Experiment 5), Mauchly’s test of sphericity was significant for phase by cue interaction, W = 0.87; χ^2^(2) = 6.45, *p* = 0.04. Therefore, we report Greenhouse–Geisser corrected values. The main effects of phase, *F*_(2, 92)_ = 0.01, *p* = 0.99, and Location *F*_(1, 46)_ = 1.54, *p* = 0.22 were not statistically significant, but the 2-way interaction was, *F*_(1.76, 81.16)_ = 7.08, *p* = 0.002, partial η^2^ = 0.13 (Figure 4a). Pairwise comparisons for the left location showed no difference in cueing between phases (baseline: M = 0.92; SE = 0.24; learning: M = 0.40; SE = 0.15; testing: M = 0.23; SE = 0.21). Similarly, for the right location there were no difference in cueing between phases (baseline: M = 0.39; SE = 0.21; learning: M = 0.87; SE = 0.16; testing: M = 1.02; SE = 0.18). However, whereas cueing for the two locations did not differ in the baseline *p* = 0.12 and learning, *p* = 0.06, phases in the testing phase cueing for the left was smaller than for the right location, *p* = 0.007. This effect was not anticipated, and possible accounts are described in the discussion.

*Response Accuracy*: The Mauchly’s Test of sphericity was significant for Phase, W = 0.69; χ^2^(2) = 16.92, *p* < 0.001, and for phase by cue, W = 0.76; χ^2^(2) = 12.26, *p* = 0.002. Therefore, we report Greenhouse–Geisser corrected values. Results showed only a significant phase by Cue interaction, *F*_(1.62, 74.29)_ = 5.51, *p* = 0.01, partial η^2^ = 0.11. Pairwise comparisons showed a significant difference in response accuracy in the testing phase, as it was greater on trials with valid cues (M = 0.96, SE = 0.05) than on trial with invalid cues (M = 0.94, SE = 0.05), *p* = 0.016 (see Figure 4b).

*Cueing Index*: For gaze cues with location bias (Experiment 6), the main effects of phase was not significant, *F*_(2, 88)_ = 0.38, *p* = 0.69 but location was significant, *F*_(1, 44)_ = 19.52, *p* < 0.001, partial η^2^ = 0.31, with larger cueing at the rich location (M = 0.92, SE = 0.15) than at the scarce location (M = −0.11, SE = 0.14). The 2-way interaction was also significant *F*_(2, 88)_ = 23.27, *p* < 0.001, partial η^2^ = 0.35, (see Figure 4c). Pairwise comparisons for the rich location showed that, unlike what was observed for arrow cues, cueing was greater in the learning phase (M = 1.39, SE = 0.19) than in the baseline (M = 0.29, SE = 0.22), p < 0.001, with a BF_10_ > 100 indicating strong evidence. It was greater in the testing phase (M = 1.09, SE = 0.23) than in the baseline, *p* = 0.02, with a BF_10_ = 5.22 indicating moderate evidence. Cueing did not differ between the learning and testing phase, *p* = 0.84, as it persisted after the regularities were removed. In contrast—and similarly to what observed in all experiments with location bias—for the scarce location cueing was smaller in the learning (M = −0.78, SE = 0.21) than in the baseline (M = 0.68, SE = 0.22), *p* < 0.001 with the BF_10_ > 100 indicating very strong evidence and it was smaller in the testing (M = −0.22, SE = 0.23) than in the baseline, *p* = 0.01, with the BF_10_ = 8.08 indicating strong evidence. Cueing did not differ between the learning and the testing phase, *p* = 0.19, again indicating that it persisted after the regularities were removed. This resulted in no differences in the baseline (*p* = 0.23), and statistically significant differences in the learning (*p* < 0.001), and testing phases (*p* < 0.001), where cueing was larger for the rich than for the scarce location. Evidence in favor of cueing differences between the two locations is very strong in both the learning (BF_10_ > 100) and testing (BF_10_ = 65.83) phases.

*Response Accuracy*: Mauchly’s test of sphericity was significant for phase, W = 0.85; χ^2^(2) = 7.11, *p* = 0.03. Therefore, we report Greenhouse–Geisser corrected values. Results showed only a significant main effect of phase *F*_(1.74, 76.36)_ = 3.47, *p* = 0.04, partial η^2^ = 0.07 (Figure 4d). Pairwise comparisons showed that response accuracy in testing (M = 0.98, SE = 0.03) was greater than in the baseline (M = 0.96, SE = 0.05), *p* = 0.016. The Cue main effect was also significant, *F*_(1, 44)_ = 4.84, *p* = 0.03, partial η^2^ = 0.10, with greater accuracy on trials with valid cues (M = 0.97, SE = 0.03) than on trials with invalid cues (M = 0.96, SE = 0.04). No other interaction or main effects reached statistical significance (see Supplementary material).

To sum up, regarding exogenous, increasing the predictive validity of gaze cues alone (Experiment 5) did not yield habitual attention. However, an unexpected laterality effect was observed, which is difficult to explain. Possible accounts for this effect are outlined in the discussion. Importantly, when cue predictive validity was paired with probability cueing, learning with gaze cues yielded a pattern of habitual attention that consisted of an advantage for the rich location and a cost for the scarce location. Next, as in all experiments cueing reflects both the effect of the cue and that of the target, in the last experiment we assess whether learning from probability cueing alone (i.e., presenting the target alone more frequently at one location) yields an advantage (i.e., faster responses) for the rich location, a cost for the scarce location (i.e., slower responses) or both. This would help to understand whether the cost observed in all experiments with location bias (Experiments 1, 3, and 5) in a scarce location is due to probability cueing.

### 3.4 Experiment 7 no cues

**Figure 5:**
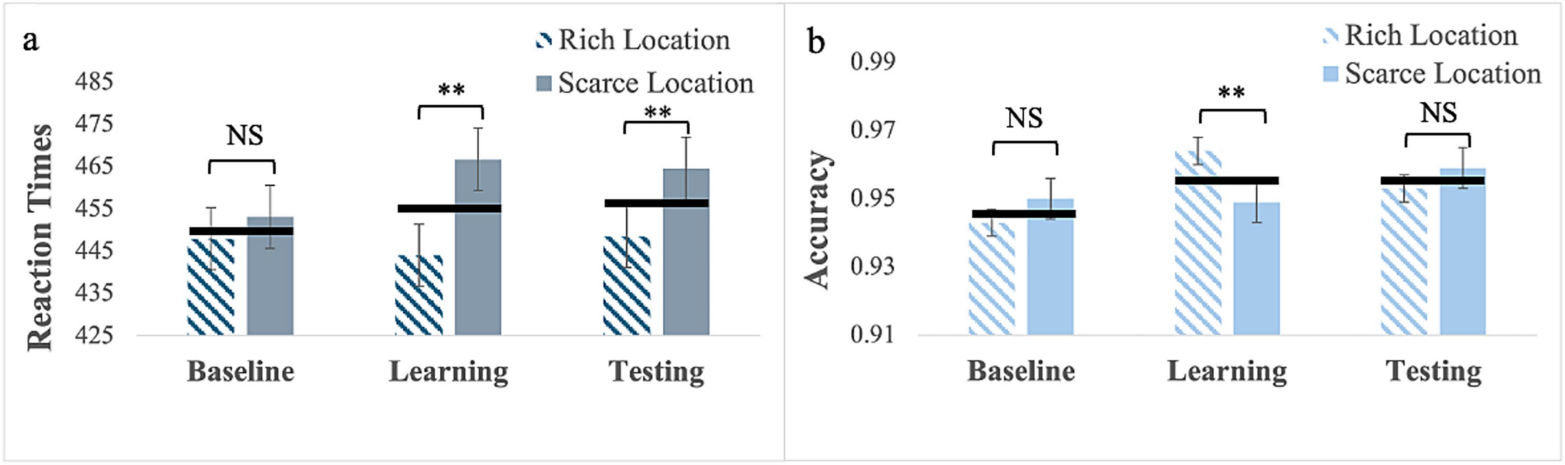
RTs **(a)** and response accuracy **(b)** for Experiment 7 with the target only presented with location bias. Error bars are standard errors of the mean. Asterisks denote significant differences: one asterisk (*) for *p* < 0.05; two asterisks (**) for *p* < 0.001. Black horizontal bars denote average values for each pair.

*RTs*: For targets (no cues) presented with location bias (Experiment 7), Mauchly’s test of sphericity was significant for phase, W = 0.63; χ^2^(2) = 20.10, *p* < 0.001. Therefore, we report Greenhouse–Geisser corrected values. The main effect of phase was not significant, *F*_(1.46, 65.85)_ = 1.27, *p* = 0.28. However, the main effect of location was, *F*_(1, 45)_ = 36.01, *p* < 0.001, partial η^2^ = 0.44, with faster RTs to targets presented at the rich location, (M = 447; SE = 6.97) than at the scarce location, (M = 461; SE = 7.38). The phase by location interaction was also significant, *F*_(2, 90)_ = 9.52, *p* < 0.001, partial η^2^ = 0.18 (see Figure 5a).

Pairwise comparisons for the rich location revealed no significant differences (baseline: M = 448, SE = 7.52; learning: M = 444, SE = 7.05; testing: M = 449, SE = 7.58). In contrast for the scarce location, RTs were longer in the learning phase (M = 467, SE = 8.05) than in the baseline (M = 453, SE = 7.55), *p* = 0.015, indicating that learning occurred and the BF_10_ = 7.05 qualifies it as strong evidence, but they did not differ between learning and testing (M = 465; SE = 8.00) phase, *p* > 0.99. This pattern resulted in no differences in RTs between the two locations in the baseline *p* = 0.07 and in statistically significant differences in learning, p < 0.001 and testing, p < 0.001. This evidence is very strong in both the learning and testing phases, with a BF_10_ > 100.

*Response Accuracy*: Mauchly’s test of sphericity was significant for phase, W = 0.82; χ2(2) = 8.87, *p* = 0.01. Therefore, we report Greenhouse–Geisser corrected values. The main effect of phase was statistically significant, *F*_(1.69, 76.11)_ = 3.89, *p* = 0.03, partial η^2^ = 0.08. Pairwise comparisons showed greater response accuracy in the learning phase (M = 0.96; SE = 0.004) than in the baseline (M = 0.95; SE = 0.007), *p* = 0.02. The 2-way interaction was also significant, *F*_(2, 90)_ = 4.81, *p* = 0.01, partial η^2^ = 0.10. Pairwise comparisons for the rich location showed that response accuracy was greater in the learning phase (M = 0.96; SE = 0.008) than in the baseline phase (M = 0.94; SE = 0.004), *p* = 0.002. In contrast, for the scarce location, there were no differences in response accuracy between the baseline (M = 0.95, SE = 0.006), learning (M = 0.95, SE = 0.006), and testing (M = 0.96, SE = 0.006) (see Supplementary material). This pattern resulted in the only significant difference being in the learning phase, *p* < 0.001, with greater accuracy for the rich location (see Figure 5b and Supplementary material).

In summary, probability cueing alone is learned, but this learning does not affect response speed to targets presented at the rich location; only response accuracy is affected. In contrast, learning based on probability cueing alone yields a cost for the scarce location as it slows down responses. Therefore, the advantage observed for the rich location occurred only with gaze cues.

## 4 Discussion

We report findings from a series of seven experiments assessing whether implicit regularities implemented in a Posner task with different types of cues are learned and guide attention even when the regularities are no longer present. More specifically, we used exogenous cues in Experiments 1 and 2, non-social (arrow) directional cues in Experiments 3 and 4, and social directional (gaze) cues in Experiments 5 and 6. Throughout the different phases, we varied cue predictive validity while probability cueing, that is, the frequency with which targets appear at the two locations was equal between locations (i.e., Experiments 1, 3, and 5), or we varied cue predictive validity and probability cueing together (i.e., Experiments 2, 4, and 6). Finally, in Experiment 7, we only varied probability cueing for the two locations. In all experiments, cues were not predictive in the baseline and testing phases (50% cue predictive validity) and were predictive (75% cue predictive validity) during the learning phase. Moreover, in all experiments, we used the SOAs known to optimize cueing effects for that specific type of cue. Although there is evidence of larger cueing effects when using dynamic face cues (McKay et al., 2021), we opted not to use a pre-cue to minimize methodological differences between experiments. This allowed us to assess whether the regularities implemented with the different cues were learned, resulting in an acquired habit that guides attention in similar ways for the different cues.

These findings revealed a clear difference in how we learn from regularities and acquire an attentional habit with these different cues, with social cues standing out from the others. In fact, whereas regularities entailed by cue predictive validity alone affected cueing effects in all cases only when they were present (i.e., in the learning phase), these regularities were learned and guided attention only when cue predictive validity was combined with probability cueing (Experiments 2, 4, and 6). Importantly, the direction of the effect of habitual attention (i.e., larger or smaller cueing effects) differed depending on the type of cue and, in line with our predictions, only regularities with social cues resulted in qualitatively different patterns of habit formation for the two locations.

More specifically, for exogenous cues, the acquired attentional habit involves a cost associated with the scarce location. Previous studies have shown that exogenous cues, such as peripheral luminance changes, can rapidly and involuntarily capture attention, especially when predictive of target location (e.g., Müller and Rabbitt, 1989; Yantis and Jonides, 1990). However, such effects tend to diminish when predictive value is removed unless accompanied by spatial biases (Theeuwes, 1994; Wang and Theeuwes, 2018), a pattern replicated in our Experiment 2, where only the combination of predictive validity and location probability led to persistent cueing effects.

Regularities with arrow cues have a somewhat different effect than predictive validity alone (Experiment 3), yielding smaller cueing effects in the learning phase, but this effect does not persist once the regularities are removed (testing phase). Although this finding is somewhat surprising, a combination of factors may be responsible for the smaller cueing effect observed. These factors rely on the inherent directional information of the arrow, the type of orientation it elicits, and the potential interference between the cue and the target (Jollie et al., 2016; Bonventre and Marotta, 2023; Tanaka et al., 2024). However, as our experiments were not designed to assess these factors, this account remains tentative. Symbolic cues such as arrows typically require voluntary orienting and engage endogenous attention (Tipples, 2002; Ristic and Kingstone, 2006). Although they can support learning of spatial regularities, prior work has suggested they may be less effective at inducing long-lasting attentional biases compared to social cues (Vecera and Rizzo, 2006). This aligns with our observation that while arrow cues influenced attention during learning, their effects did not persist consistently once the regularities were removed. In contrast, when cue predictive validity and probability cueing (rich and scarce location) were combined in Experiment 4, these regularities did not induce a habit formation for the rich location but similarly to the pattern observed with exogenous cues yield habitual attention with a cost for the scarce location, as cueing effects were smaller when regularities were present (i.e., learning phase), as well as after they were removed (i.e., testing phase). In this case, there was also a cost in terms of reduced response accuracy, but it was present only in the learning phase, suggesting a temporary monitoring adjustment, as discussed earlier.

Finally, when regularities were implemented with gaze, cueing effects were similar for the two locations (i.e., left and right) in both the baseline and learning phases. However, there was an unpredicted laterality effect in the testing phase, with smaller cueing for the left location. This finding is at odds with the right hemisphere lateralization observed for processing and orienting attention to schematic, non-predictive, gaze cues, as observed in the baseline and testing phases of the present experiment (which should yield greater cueing for the contralateral, left location) (Marotta et al., 2012). The advantage for the right location (left hemisphere) was present only during the testing phase, suggesting that both hemispheres are involved in volitionally orienting attention to gaze direction. However, only one hemisphere is involved in orienting attention reflexively to gaze direction (Kingstone et al., 2000). Accordingly, if this lateralization effect is replicated in future studies, it could suggest that learned attentional habit with gaze cues is lateralized to the left hemisphere, which has been linked to controlling responses to familiar and routine situations (as in the case of habitual attention) (Kingstone et al., 2000). Since this effect was not anticipated, this account remains tentative, and future research could help determine whether it is reliable when controlling for individuals’ hemispheric specialization and manual dominance, possibly by monitoring eye movements. In fact, although we used a chinrest to reduce head movements and task instructions stressed the importance of fixating the central plus sign at the beginning of each trial, we did not monitor eye movements in the present study, leaving open the possibility that the laterality effects observed could be due to uncontrolled eye position at the beginning of each trial. However, if this were the case, then it would apply to all experiments. When the predictive validity of gaze cues was combined with probability cueing in Experiment 6 with location bias, findings were clear, showing strong evidence that regularities in gaze direction enhanced cueing effects for the rich location (i.e., an advantage) and reduced cueing effects for the scarce location (i.e., a cost). Importantly, these effects occurred when the regularities were present for both locations, as well as after they were removed, with Bayesian analyses showing moderate evidence for the rich location and strong evidence for the scarce location.

We had anticipated that learning with gaze cues would be different than learning with other cues. This is because gaze direction is uniquely social and biologically salient (Friesen and Kingstone, 1998; Driver et al., 1999). Neurocognitive evidence suggests that gaze cues engage distinct systems involving theory of mind and social cognition (Salera et al., 2024; Pelphrey et al., 2003; Nummenmaa and Calder, 2009), which may support deeper encoding of environmental regularities. Our Experiments 5 and 6 showed that participants not only learned from regularities with gaze cues but also developed qualitatively different attentional habits for rich and scarce locations, even after contingencies were removed, suggesting a robust and possibly socially reinforced attentional habit specific to social cues. Moreover, the observed effects of learning regularities on attentional orienting consisted of altering the costs/benefits engendered by the cues in terms of response speed but not in terms of response accuracy, as differences in accuracy were observed only in the learning phase in Experiment 4 with arrow cues and biased location, as discussed earlier.

Having established this, regardless of cue type, predictive validity alone does not induce an attentional habit that persists after this regularity is removed; our findings clearly show that an attentional habit can be induced by combining cue predictive validity and probability cueing. However, when this is done with exogenous and non-social arrow cues, the learned attentional habit primarily incurs a cost (i.e., reduced cueing) for the scarce location. This pattern aligns with findings from spatial statistical learning paradigms, where attentional suppression is observed for less frequently reinforced locations (Failing et al., 2019). Importantly, here we showed that attentional suppression of low-probability regions is more robust than facilitation of high-probability ones (Failing et al., 2019; Ferrante et al., 2018). In contrast, with social gaze cues, the learned attentional habit consists of an advantage for the rich location and a cost for the scarce location. Taken together, these findings suggest that habitual attention can be learned from regularities with social cues, and the effects on attention of this learning are qualitatively different from those observed with non-social cues. As the advantage for the rich location does not occur for the other cues, it is likely to be specific to gaze direction, possibly due to the social and motivational significance attributed to gaze direction (Bayliss et al., 2006). However, a challenge to such an account would be if learning based on probability cueing alone elicited an advantage for the rich location. Importantly, the results from Experiment 7, which used probability cueing alone, showed that the advantage, meaning faster responses to targets presented at the rich location, does not persist in the testing phase, when probability cueing is removed. In contrast, the cost of slower responses to targets presented at the scarce location is learned and persists to the testing phase. Therefore, we extend prior research on location probability learning and cueing effects by demonstrating that different types of cues differentially support the formation of enduring attentional habits. Importantly, whereas there is ample evidence that we acquire preferences based on regularities between the gaze direction of another person and the object they look at, as we learn that people look at what they like (Bayliss et al., 2006; Tipples and Pecchinenda, 2019; Einav and Hood, 2006). Here, we provide the first evidence that regularities with gaze direction are learned and induce an attentional habit. Interestingly, this type of learning is preserved in old age (Salera et al., 2024).

We should acknowledge some limitations of the present findings. Firstly, in the experiments with gaze cues, a schematic face was used, which one could argue has limited ecological validity. While we recognize the importance of enhancing the ecological validity of the stimuli used, we also note that Dalmaso et al. (2025) have recently compared the cueing effects elicited by schematic and real faces, concluding that there are no substantial differences. Most importantly, learning effects similar to those reported here were also observed by Salera et al. (2024), who used real face pictures looking left or right with older individuals. Finally, our findings revealed two unexpected effects: one in Experiment 4, where location bias was observed (Figure 3c), resulting in smaller cueing effects for the predictive validity of arrow cues; the other in Experiment 5, where no location bias was observed (Figure 4a), leading to smaller cueing effects for the testing phase in the left location. As discussed earlier, we have put forward some tentative explanations for future research to assess.

The present study offers a novel contribution by systematically disentangling the effects of cue predictive validity and spatial probability cueing across social, symbolic, and exogenous cues within the same experimental framework. Unlike prior research, which often examined these factors in isolation or focused on a single cue type, our seven experiments allowed for a systematic comparison of how different cues support the formation of attentional habits. Critically, we demonstrated that only gaze cues, due to their social and motivational salience, produce a robust, bidirectional attentional habit that persists even after regularities are removed. This effect was not observed with exogenous or arrow cues, even when combined with spatial bias, highlighting a qualitative difference in how social signals shape long-term attentional patterns. Methodologically, the use of tightly matched cueing paradigms, uniform manipulations of contingency phases, and inclusion of both learning and extinction phases provides a rigorous basis for isolating the mechanisms of attentional habit formation across cue types.

## Data Availability

The datasets presented in this study can be found in online repositories. The names of the repository/repositories and accession number(s) can be found in the article/Supplementary material.
