# Supplementary material for: Social and non-social directional cues differentially orient attention by learned habit
Source: Front Hum Neurosci. 2025 Sep 9;19:1636726. doi: 10.3389/fnhum.2025.1636726 (PMC12454303; doi:10.3389/fnhum.2025.1636726)
Supplement: Supplementary file 1 [file Table_1.DOCX]

**Supplementary Material**

*Exp. 1 - exogenous cues without location bias*

*Reaction times (see Table 1S)*: For exogenous cues without location bias (experiment 1), the Mauchly’s Test of sphericity was significant for Phase, W= .58; χ2(2) = 21.25, *p* < .001, Phase by Cue, W= .77; χ2(2) = 9.98, *p* = .007, and Phase by Cue by Location interaction, W= .76; χ2(2) = 10.62, *p* = .005. Therefore, we report Greenhouse-Geisser corrected values. There was a significant main effect of Phase, F_(1.41, 56.33)_= 7.62, *p*=.003, partial η² =.16. Pairwise comparisons showed faster RTs during testing (M=504; SE=6.39) compared to the baseline (M=519; SE=6.91; *p*=.011) and learning (M=512; SE=6.04; *p*=.016), but no differences between baseline and learning (*p*=.19).

The main effect of Cue was also significant, F(_1, 40)_= 254.1, *p*<.001, partial η² =.86, with faster RT to valid trials (M= 493, SE= 6.15) compared to invalid trials (M= 530, SE= 6.17).

The effect of Location was not significant, F_(1, 40)_= .26, *p*=.61, but it was the 2-way Phase by Cue interaction, F_(1.63, 65.26)_= 3.72, *p*=.038, partial η² =.09, due to generally faster RTs for valid trials (baseline: M= 503, SE= 7.0; learning: M= 491, SE= 6.33, testing: M= 485, SE= 6.48), compared to invalid trials (baseline: M= 535, SE= 7.12; learning: M= 532, SE= 6.05, testing: M= 523, SE= 6.7), in each phase (*p*<.001 for all comparisons).

Phase by Location, F_(2, 80)_= 1.26, *p*=.29, Cue by Location, F_(1, 40)_< .001, *p*=.98, and Phase by Cue by Location interactions, F_(1.62, 64.61)_= 2.11, *p*=.14, were not significant.

| PHASE | CUE | LOCATION | MEAN | SE |
| --- | --- | --- | --- | --- |
| Baseline | Valid | Left | 503 | 7.54 |
|  |  | Right | 503 | 6.99 |
|  | Invalid | Left | 537 | 6.8 |
|  |  | Right | 534 | 7.85 |
| Learning | Valid | Left | 490 | 6.35 |
|  |  | Right | 493 | 6.57 |
|  | Invalid | Left | 534 | 6.15 |
|  |  | Right | 531 | 6.36 |
| Testing | Valid | Left | 485 | 6.26 |
|  |  | Right | 484 | 7.54 |
|  | Invalid | Left | 519 | 6.62 |
|  |  | Right | 527 | 7.34 |

Table 1S. Reaction times in Experiment 1 across all Phase, Cue and Location conditions.

*Response Accuracy (see Table 2S)*: The Mauchly's Test of sphericity was significant for Phase by Cue, W= .65; χ2(2) = 16.56, *p* < .001, Phase by Location, W= .85; χ2(2) = 6.49, *p* = .039, and Phase by Cue by Location, = .62; χ2(2) = 18.44, *p* < .001. Therefore, we report Greenhouse-Geisser corrected values. Results showed only a significant main effect of Phase F_(2, 80)_= 3.61, *p*=.032, partial η² =.083. However, pairwise comparisons showed no significant differences across phases (baseline: M= .978, SE= .004; learning: M= .981, SE= .003, testing: M= .984, SE= .002), baseline vs. learning p=.522, baseline vs. testing *p*=.075, learning vs. testing *p*=.378. The main effects of Cue, F(_1, 40)_= .18, *p*=.893, and Location, F(_1, 40)_= .10, *p*=.919, and the Phase by Cue, F_(1.49, 59.44)_= .68, *p*=.47, Phase by Location, F_(1.73, 69.36)_= 1.12, *p*=.47, Cue by Location, F_(1, 40)_= 2.47, *p*=.124, and Phase by Cue by Location, F_(1.45, 58.11)_= 1.37, *p*=.258, interactions did not reached statistical significance.

| PHASE | CUE | LOCATION | MEAN | SE |
| --- | --- | --- | --- | --- |
| Baseline | Valid | Left | .981 | .005 |
|  |  | Right | .975 | .007 |
|  | Invalid | Left | .979 | .005 |
|  |  | Right | .976 | .006 |
| Learning | Valid | Left | .982 | .003 |
|  |  | Right | .983 | .003 |
|  | Invalid | Left | .979 | .004 |
|  |  | Right | .981 | .004 |
| Testing | Valid | Left | .986 | .004 |
|  |  | Right | .979 | .004 |
|  | Invalid | Left | .981 | .004 |
|  |  | Right | .991 | .003 |

Table 2S. Accuracies in Experiment 1 across all Phase, Cue and Location conditions.

Experiment 2 - *exogenous cues with location bias*

*Reaction times (see Table 3S)*: For exogenous cues with location bias (experiment 2), the Mauchly's Test of sphericity was significant for Phase, W= .83; χ2(2) = 8.24, *p* = .016, therefore we report Greenhouse-Geisser corrected values. There was a significant main effect of Phase, F(_1.70, 74.93)_= 8.74, *p*<.001, partial η² =.17, due to faster RTs during testing (M=501, SE=5.9) compared to learning (M=510, SE=6.4; *p*=.005) and baseline (M=515, SE=6.1; *p*=.004). The main effect of Cue was significant, F(_1, 44)_= 148.58, *p*<.001, partial η² =.77, due to faster RT for valid trials (M=499, SE=5.8) compared to invalid trials (M=518, SE=5.97). Location was significant, F_(1, 44)_= 27.04, *p*<.001, partial η² =.38, with faster RT for targets at the rich location (M= 505, SE= 5.9) than at the scarce location (M= 513, SE= 5.87). The Phase by Cue (F_(2, 88)_= 35.47, *p*<.001, partial η² =.45), Phase by Location (F_(2, 88)_= 67.75, *p*<.001, partial η² =.61), and Cue by Location (F(_1, 44)_= 26.91, *p*<.001, partial η² =.38) interactions were significant. Finally, the Phase by Cue by Location interaction was significant (F_(2, 88)_= 13.07, *p*<.001, partial η² =.23). Pairwise comparisons showed that RTs were faster for valid trials for all phases and locations, compared to invalid trials (p<.001 for all comparisons). Moreover, significant differences were found between valid trials in the rich (M= 483, SE= 6.15) and scarce (M= 536, SE= 7.59) location during learning (p<.001), between invalid trials in the rich (M= 506, SE= 6.3) and scarce (M= 517, SE= 6.88) location during learning (p<.001), and between invalid trials in the rich (M= 522, SE= 6.35) and scarce (M= 507, SE= 6.93) location during testing (p<.001).

| PHASE | CUE | LOCATION | MEAN | SE |
| --- | --- | --- | --- | --- |
| Baseline | Valid | Rich | 502 | 7.23 |
|  |  | Scarce | 499 | 5.71 |
|  | Invalid | Rich | 532 | 6.2 |
|  |  | Scarce | 527 | 6.79 |
| Learning | Valid | Rich | 483 | 6.14 |
|  |  | Scarce | 536 | 7.59 |
|  | Invalid | Rich | 506 | 6.3 |
|  |  | Scarce | 517 | 6.88 |
| Testing | Valid | Rich | 487 | 6.7 |
|  |  | Scarce | 489 | 5.59 |
|  | Invalid | Rich | 522 | 6.35 |
|  |  | Scarce | 507 | 6.93 |

Table 3S. Reaction times in Experiment 2 across all Phase, Cue and Location conditions.

*Response Accuracy (see Table 4S)*: Results showed that the main effects of Phase, F(_2, 88)_= .11, *p*=.895, Cue, F(_1, 44)_= .003, *p*=.959, and Location were not significant, F_(1, 44)_= .3, *p*=.585. The Phase by Cue (F_(2, 88)_= .83, *p*=.44), Phase by Location (F(_2, 88)_= .46, *p*=.633), Cue by Location (F(_1, 44)_= .98, *p*=.327) and Phase by Cue by Location interactions were not significant (F_(2, 88)_= .6, *p*=.553).

| PHASE | CUE | LOCATION | MEAN | SE |
| --- | --- | --- | --- | --- |
| Baseline | Valid | Rich | .975 | .005 |
|  |  | Scarce | .977 | .004 |
|  | Invalid | Rich | .980 | .004 |
|  |  | Scarce | .979 | .004 |
| Learning | Valid | Rich | .981 | .003 |
|  |  | Scarce | .980 | .003 |
|  | Invalid | Rich | .973 | .007 |
|  |  | Scarce | .981 | .003 |
| Testing | Valid | Rich | .981 | .004 |
|  |  | Scarce | .977 | .004 |
|  | Invalid | Rich | .978 | .006 |
|  |  | Scarce | .980 | .004 |

Table 4S. Accuracies in Experiment 2 across all Phase, Cue and Location conditions.

*Exp. 3 – Arrow cues without location bias*

*Reaction times (see Table 5S)*: For arrow cues without location bias (experiment 3), the Mauchly's Test of sphericity was significant for Phase, W= .63; χ2(2) = 22.94, *p* < .001. Therefore, we report Greenhouse-Geisser corrected values. There was a significant main effect of Phase, F(_1.46, 72.79)_= 6.16, *p*=.008, partial η² =.11. Pairwise comparisons showed faster RTs during testing (M=463; SE=6.94) compared to the baseline (M=477; SE=7.79; *p*=.026) and learning (M=472; SE=6.99; *p*=.033), but no differences between baseline and learning *(p*=.404).

The main effect of Cue was also significant, F(_1, 50)_= 41.63, *p*<.001, partial η² =.45, with faster RT to valid trials (M= 465, SE= 6.75) compared to invalid trials (M= 477, SE= 7.13), *p*<.001.

Location was not significant, but it was the 2-way Phase by Cue interaction, F_(2, 100)_= 19.82, *p*<.001, partial η² =.28, due to generally faster RTs for valid compared to invalid trials in the baseline (valid: M= 467, SE= 7.74; invalid: M= 487, SE= 8.04, *p*<.001) and testing phases (valid: M= 455, SE= 6.83; invalid: M= 472, SE= 7.41, *p*<.001) compared to invalid trials, in each phase (*p*<.001 for all comparisons), but not during learning (valid: M= 473, SE= 7.2; invalid: M= 471, SE= 6.97, *p*=.531).

The Phase by Location, F_(2, 100)_= 1.26, *p*=.288, Cue by Location, F_(1, 50)_= 2.63, *p*=.111,, and Phase by Cue by Location interactions were not significant, F_(2, 100)_= 2.77, *p*=.068,.

| PHASE | CUE | LOCATION | MEAN | SE |
| --- | --- | --- | --- | --- |
| Baseline | Valid | Left | 469 | 8.05 |
|  |  | Right | 466 | 7.95 |
|  | Invalid | Left | 485 | 8.63 |
|  |  | Right | 489 | 7.85 |
| Learning | Valid | Left | 473 | 7.0 |
|  |  | Right | 473 | 7.61 |
|  | Invalid | Left | 474 | 6.95 |
|  |  | Right | 469 | 7.23 |
| Testing | Valid | Left | 457 | 6.75 |
|  |  | Right | 453 | 7.25 |
|  | Invalid | Left | 468 | 7.58 |
|  |  | Right | 476 | 7.7 |

Table 5S. Reaction times in Experiment 3 across all Phase, Cue and Location conditions.

*Response Accuracy (see Table 6)*: The Mauchly's Test of Sphericity was significant for Phase, W= .67; χ2(2) = 19.44, *p* < .001. Therefore, we report Greenhouse-Geisser corrected values. Results showed that the main effects of Phase, F(_1.51, 75.33)_= 1.02, *p*=.364, Cue, F(_1, 50)_= 2.1, *p*=.154, and Location were not significant, F_(1, 50)_= .09, *p*=.769. The Phase by Cue (F_(2, 100)_= 1.67, *p*=.193), Phase by Location (F(_2, 100)_= 1.84, *p*=.163), Cue by Location (F(_1, 50)_= .02, *p*=.899) and Phase by Cue by Location interactions were not significant (F_(2, 100)_= 1.17, *p*=.315).

| PHASE | CUE | LOCATION | MEAN | SE |
| --- | --- | --- | --- | --- |
| Baseline | Valid | Left | .958 | .006 |
|  |  | Right | .956 | .006 |
|  | Invalid | Left | .957 | .006 |
|  |  | Right | .948 | .009 |
| Learning | Valid | Left | .947 | .007 |
|  |  | Right | .950 | .007 |
|  | Invalid | Left | .946 | .007 |
|  |  | Right | .958 | .007 |
| Testing | Valid | Left | .953 | .008 |
|  |  | Right | .953 | .007 |
|  | Invalid | Left | .943 | .011 |
|  |  | Right | .944 | .008 |

Table 6S. Accuracies in Experiment 3 across all Phase, Cue and Location conditions.

Experiment 4 - *Arrow cues with location bias*

*Reaction times (see Table 7S)*: For arrow cues with location bias (experiment 4), the Mauchly's Test of sphericity was significant for Phase, W= .64; χ2(2) = 19.04, p < .001, and Phase by Cue by Location interaction, W= .75; χ2(2) = 12.63, p = .002. Therefore, we report Greenhouse-Geisser corrected values. There was a significant main effect of Phase, F(_1.47, 64.81)_= 7.307, *p*=.004, partial η² =.14, was significant, due to faster RT during testing (M=515, SE=8.85) compared to the baseline (M=529, SE=9.69; *p*=.012). The main effect of Cue was significant, F(_1, 44)_= 28.04, *p*<.001, partial η² =.39, due to faster RT for valid trials (M=518, SE=8.84) compared to invalid trials (M=527, SE=9.27), p<.001. Location, F(_1, 44)_= .54, *p*=.465, was not significant. The Phase by Cue interaction was significant (F_(2, 88)_= 14.35, *p*<.001, partial η² =.25), as well as Phase by Location (F(_2, 88)_= 5.63, *p*=.005, partial η² =.11), and Cue by Location interactions (F(_1, 44)_= 15.19, *p*<.001, partial η² =.26) were significant. Finally, the Phase by Cue by Location interaction was significant (F(_1.59, 70.15)_= 10.49, *p*<.001, partial η² =.19). Pairwise comparisons showed that RTs were faster for valid trials for all phases and locations, compared to invalid trials (*p*<.001 for all comparisons), except for the scarce location (*p*=.65). Moreover, significant differences were found between valid trials in the rich (M= 509, SE= 9.27) and scarce (M= 535, SE= 9.46) location during learning (*p*<.001), between invalid trials in the rich (M= 528, SE=9.06) and scarce (M= 516, SE= 10.43) location during learning (*p*=.023), and between invalid trials in the rich (M= 528, SE= 9.85) and scarce (M= 513, SE= 9.26) location during testing (*p*=.005).

| PHASE | CUE | LOCATION | MEAN | SE |
| --- | --- | --- | --- | --- |
| Baseline | Valid | Rich | 520 | 9.63 |
|  |  | Scarce | 521 | 9.87 |
|  | Invalid | Rich | 536 | 9.86 |
|  |  | Scarce | 541 | 10.8 |
| Learning | Valid | Rich | 509 | 9.27 |
|  |  | Scarce | 535 | 9.46 |
|  | Invalid | Rich | 528 | 9.06 |
|  |  | Scarce | 516 | 10.43 |
| Testing | Valid | Rich | 509 | 9.58 |
|  |  | Scarce | 511 | 8.31 |
|  | Invalid | Rich | 528 | 9.85 |
|  |  | Scarce | 513 | 9.26 |

Table 7S. Reaction times in Experiment 4 across all Phase, Cue and Location conditions.

*Response Accuracy (see Table 8S)*: the Mauchly's Test of sphericity was significant for Phase, W= .78; χ2(2) = 10.55, *p* =.005, and Phase by Cue interaction, W= .74; χ2(2) = 12.72, *p* = .002. Therefore, we report Greenhouse-Geisser corrected values. Results showed that the main effects of Phase, F(_1.64, 70.36)_= 3.0, *p*=.066, Cue, F(_1, 43)_= .2, *p*=.653, and Location were not significant, F_(1, 43)_= .005, *p*=.945. In contrast, the Phase by Cue interaction was significant, (F_(1.59, 68.18)_= 3.54, *p*=.045, partial η² =.08). Responses in trials with invalid cues (M=.097, SE=.005) were more accurate than those with valid cues (M=.096, SE=.005) during the learning phase. Accuracies did not differ as a function of cue validity in the baseline (valid: M=.097, SE=.006; invalid M=.096, SE=.006, *p*=.215) and in the testing phases (valid: M=.097, SE=.005; invalid M=.097, SE=.005, *p*=.231). The Phase by Location (F(_2, 86)_= 1.27, *p*=.285), Cue by Location (F(_1, 43)_= .1.82, *p*=.184) and Phase by Cue by Location interactions were not significant (F_(2, 86)_= .43, *p*=.652).

| PHASE | CUE | LOCATION | MEAN | SE |
| --- | --- | --- | --- | --- |
| Baseline | Valid | Rich | .965 | .007 |
|  |  | Scarce | .964 | .006 |
|  | Invalid | Rich | .950 | .008 |
|  |  | Scarce | .963 | .006 |
| Learning | Valid | Rich | .962 | .006 |
|  |  | Scarce | .959 | .006 |
|  | Invalid | Rich | .971 | .005 |
|  |  | Scarce | .969 | .005 |
| Testing | Valid | Rich | .976 | .005 |
|  |  | Scarce | .970 | .006 |
|  | Invalid | Rich | .968 | .007 |
|  |  | Scarce | .969 | .006 |

Table 8S. Accuracies in Experiment 4 across all Phase, Cue and Location conditions.

*Exp. 5 – Gaze cues without location bias*

*Reaction times (see Table 9S)*: For gaze cues without location bias (experiment 5), the Mauchly's Test of sphericity was significant for Phase, W= .795; χ2(2) = 10.3, p= .006, Phase by Location, W= .87; χ2(2) = 6.26, p= .044, and Phase by Cue by Location, W= .85; χ2(2) = 7.14, p= .028. Therefore, we report Greenhouse-Geisser corrected values. There was a significant main effect of Phase, F(_1.66, 76.38)_= 16.32, *p*<.001, partial η² =.26. Pairwise comparisons showed faster RTs during testing (M=454; SE=6.1) compared to the baseline (M=472; SE=6.66; *p*<.001) and learning (M=465; SE=6.05; *p*<.001), but no differences between baseline and learning (*p*=.09).

The main effect of Cue was also significant, F(_1, 46)_= 56.37, *p*<.001, partial η² =.55, with faster RT to valid trials (M= 457, SE= 5.7) compared to invalid trials (M= 469, SE= 6.4), *p*<.001.

The main effect of Location, F(_1, 46)_= .19, *p*=.667, as well as the Phase by Cue, F(_2, 92)_= .05, *p*=.995, Phase by Location, F(_1,.77, 81.43)_= .28, *p*=.729, and Cue by Location, F(_1, 46)_= 1.17, *p*=.285 interactions were not significant, but it was the Phase by Cue by Location interaction, F(_1.74, 80.23)_= 8.41, *p*<.001, partial η² =.16. Pairwise comparisons showed that RTs were generally faster for valid trials, compared to invalid trials (baseline, left: *p*<.001; learning, left: *p*=.013, right: *p*<.001; testing, right: *p*<.001), except for the right location during the baseline (*p*=.08) and the left location during testing (*p*=.25).

| PHASE | CUE | LOCATION | MEAN | SE |
| --- | --- | --- | --- | --- |
| Baseline | Valid | Left | 462 | 6.12 |
|  |  | Right | 468 | 7.18 |
|  | Invalid | Left | 481 | 8.43 |
|  |  | Right | 475 | 6.55 |
| Learning | Valid | Left | 461 | 6.23 |
|  |  | Right | 456 | 6.01 |
|  | Invalid | Left | 469 | 6.43 |
|  |  | Right | 472 | 6.4 |
| Testing | Valid | Left | 450 | 6.17 |
|  |  | Right | 445 | 5.69 |
|  | Invalid | Left | 455 | 6.68 |
|  |  | Right | 464 | 7.21 |

Table 9S. Reaction times in Experiment 5 across all Phase, Cue and Location conditions.

*Response Accuracy (see Table 10S)*: the Mauchly's Test of sphericity was significant for Phase, W= .69; χ2(2) = 16.92, *p* <.001, and Phase by Cue interaction, W= .76; χ2(2) = 12.26, *p* = .002. Therefore, we report Greenhouse-Geisser corrected values. Results revealed that the main effects of Phase, F(_1.52, 70.05)_= 1.34, *p*=.267, Cue, F(_1, 46)_= .8, *p*=.377, and Location were not significant, F_(1, 46)_= .45, *p*=.506. The Phase by Cue interaction, (F_(1.62, 74.29)_= 5.51, *p*=.01, partial η² =.11), with pairwise comparisons showing greater accuracy for valid trials (M=.96, SE=.005) compared to invalid trials (M=.94, SE=.005) during testing (*p*=.016), but not during baseline (*p*=.10) and learning (*p*=.26). The Phase by Location (F(_2, 92)_= 2.22, *p*=.114), Cue by Location (F(_1, 46)_< .001, *p*>.999) and Phase by Cue by Location interactions were not significant (F_(2, 92)_= .59, *p*=.556).

| PHASE | CUE | LOCATION | MEAN | SE |
| --- | --- | --- | --- | --- |
| Baseline | Valid | Left | .947 | .008 |
|  |  | Right | .931 | .009 |
|  | Invalid | Left | .952 | .008 |
|  |  | Right | .945 | .008 |
| Learning | Valid | Left | .951 | .004 |
|  |  | Right | .952 | .005 |
|  | Invalid | Left | .949 | .005 |
|  |  | Right | .946 | .007 |
| Testing | Valid | Left | .954 | .006 |
|  |  | Right | .962 | .005 |
|  | Invalid | Left | .943 | .005 |
|  |  | Right | .945 | .008 |

Table 10S. Accuracies in Experiment 5 across all Phase, Cue and Location conditions.

Experiment 6 - *Gaze cues with location bias*

*Reaction times (see Table 11S)*: For gaze cues with location bias (experiment 6), the Mauchly's Test of sphericity was significant for Phase, W= .77; χ2(2) = 11.47, *p* = .003, and Phase by Cue by Location interaction, W= .84; χ2(2) = 7.5, *p* = .024. Therefore, we report Greenhouse-Geisser corrected values. There was a significant main effect of Phase, F(_1.62, 71.31)_= 6.94, *p*=.003, partial η² =.14, was significant, due to faster RT during testing (M=520, SE=10.4) compared to the baseline (M=536, SE=9.42; *p*=.012) and during learning (M=526, SE=8.62) compared to the baseline (*p*=.044). The main effect of Cue was significant, F(_1, 44)_= 8.83, *p*=.005, partial η² =.17, due to faster RT for valid trials (M=525, SE=9.37) compared to invalid trials (M=530, SE=8.99), *p*=.005. Location was not significant. The Phase by Cue interaction was significant (F_(2, 88)_= 7.81, *p*<.001, partial η² =.15), due to faster RT for valid trials compared to invalid trials in all phases (baseline: *p*=.002; learning: *p*=.024; testing: *p*=.012).

Phase by Location, F_(2, 88)_= 2.14, *p*=.124 and Cue by Location, F_(1, 44)_= .71, *p*=.404, interactions were not significant, but it was the Phase by Cue by Location interaction (F(_1.72, 75.86)_= 7.01, *p*=.003, partial η² =.14). Pairwise comparisons showed that RTs were faster for valid trials for all phases and locations, compared to invalid trials, except for the rich location during baseline (*p*=.15) and learning (*p*=.08), and for the scarce location during learning (p=.89) and testing (*p*=.27). Moreover, significant differences were found between invalid trials in the rich (M= 518, SE=8.81) and scarce (M= 529, SE= 8.63) location during learning (*p*=.021), between valid trials in the rich (M= 508, SE= 11.11) and scarce (M= 523, SE= 11.1) location during testing (*p*<.001), and between invalid trials in the rich (M= 530, SE= 10.45) and scarce (M= 518, SE= 10.22) location during testing (*p*=.01).

| PHASE | CUE | LOCATION | MEAN | SE |
| --- | --- | --- | --- | --- |
| Baseline | Valid | Rich | 533 | 9.1 |
|  |  | Scarce | 529 | 10.33 |
|  | Invalid | Rich | 540 | 10.43 |
|  |  | Scarce | 543 | 9.25 |
| Learning | Valid | Rich | 527 | 9.41 |
|  |  | Scarce | 529 | 9.56 |
|  | Invalid | Rich | 518 | 8.8 |
|  |  | Scarce | 529 | 8.63 |
| Testing | Valid | Rich | 508 | 11.11 |
|  |  | Scarce | 523 | 11.1 |
|  | Invalid | Rich | 530 | 10.45 |
|  |  | Scarce | 518 | 10.22 |

Table 11S. Reaction times in Experiment 6 across all Phase, Cue and Location conditions.

*Response Accuracy (see Table 12S)*: the Mauchly's Test of sphericity was significant for Phase, W= .85; χ2(2) = 7.11, *p* =.029. Therefore, we report Greenhouse-Geisser corrected values. Results showed a significant effect of Phase (F_(1.74, 76.36)_= 3.47, *p*=.042, partial η² =.08), due to greater accuracy during testing (M=.98; SE=.003) compared to baseline (M=.96; SE=.005), p=.016. The main effect of Cue was also significant (F_(1, 44)_= 4.84, *p*=.033, partial η² =.099), due to greater accuracy for valid trials (M=.97; SE=.003) compared to invalid trials (M=.96; SE=.004), *p*=.033. The main effect of Location, F_(1, 44)_= 1.3, *p*=.260, as well as the Phase by Cue (F_(2, 88)_= 1.45, *p*=.240), Phase by Location (F(_2, 88)_= .28, *p*=.756), Cue by Location (F(_1, 44)_= .03, *p*=.868) and Phase by Cue by Location, (F_(2, 88)_= .11, *p*=.898), interactions were not statistically significant.

| PHASE | CUE | LOCATION | MEAN | SE |
| --- | --- | --- | --- | --- |
| Baseline | Valid | Rich | .963 | .006 |
|  |  | Scarce | .965 | .006 |
|  | Invalid | Rich | .961 | .007 |
|  |  | Scarce | .961 | .007 |
| Learning | Valid | Rich | .971 | .004 |
|  |  | Scarce | .971 | .004 |
|  | Invalid | Rich | .956 | .008 |
|  |  | Scarce | .959 | .008 |
| Testing | Valid | Rich | .972 | .005 |
|  |  | Scarce | .979 | .004 |
|  | Invalid | Rich | .971 | .005 |
|  |  | Scarce | .975 | .005 |

Table 12S. Accuracies in Experiment 6 across all Phase, Cue and Location conditions.

Experiment 7 – *Target only*

*Reaction times (see Table 13S)*: for targets presented with location bias (experiment 7), the Mauchly's Test of sphericity was significant for Phase, W= .63; χ2(2) = 20.10, *p* < .001, therefore we report Greenhouse-Geisser corrected values. The main effect of Phase was not significant, but the main effect of Location was, F(1, 45)= 36.01, *p*<.001, partial η² =.44, with faster RTs to targets presented at the rich location, (M=447; SE= 6.97) than at the scarce location, (M= 461; SE= 7.38), *p*<.001. The Phase by Location interaction was also significant, F(2, 90)= 9.52, *p*<.001, partial η² =.18. Pairwise comparisons for the rich location showed no significant differences (baseline: M= 448; SE= 7.52; learning: M= 444; SE= 7.05; testing: M= 449; SE= 7.58). In contrast, for the scarce location, RTs were longer in the learning phase (M= 467, SE= 8.05) than in the baseline (M= 453, SE= 7.55), *p*= .015, , but they did not differ between learning and testing (M= 465; SE= 8.00) phase, *p*> .99. This pattern resulted in no differences in RTs between the two locations in the baseline p= .07 and in statistically significant differences in learning, *p*< .001 and testing, *p*< .001.

| PHASE | LOCATION | MEAN | SE |
| --- | --- | --- | --- |
| Baseline | Rich | 448 | 7.52 |
|  | Scarce | 453 | 7.55 |
| Learning | Rich | 444 | 7.01 |
|  | Scarce | 467 | 8.05 |
| Testing | Rich | 449 | 7.58 |
|  | Scarce | 465 | 7.99 |

Table 13S. Reaction times in Experiment 7 across all Phase and Location conditions.

*Response Accuracy (see Table 14S)*: The Mauchly's Test of sphericity was significant for Phase, W= .82; χ2(2) = 8.87, *p* = .01, therefore we report Greenhouse-Geisser corrected values. The main effect of Phase was statistically significant, F(1.69, 76.11)= 3.89, *p*=.03, partial η² =.08. Pairwise comparisons showed greater response accuracy in the learning phase (M= .96; SE= .004) than in the baseline (M=.95; SE= .007), *p*= .02. The 2-way interaction was also significant, F(2, 90)= 4.81, *p*=.01, partial η² =.10. Pairwise comparisons for the rich location showed that response accuracy was greater in the learning phase (M= .96; SE= .008) than in the baseline phase (M= .94; SE= .004), *p*= .002. In contrast, for the scarce location there were no differences in response accuracy between the baseline, M= .95; SE= .006, learning, M= .95; SE= .006, and testing, M= .96: SE= .006.

| PHASE | LOCATION | MEAN | SE |
| --- | --- | --- | --- |
| Baseline | Rich | .943 | .008 |
|  | Scarce | .95 | .006 |
| Learning | Rich | .964 | .004 |
|  | Scarce | .949 | .006 |
| Testing | Rich | .953 | .007 |
|  | Scarce | .959 | .006 |

Table 14S. Accuracies in Experiment 7 across all Phase and Location conditions.
